# A Systematic Assessment of MHC Class II Peptide Binding Predictions and Evaluation of a Consensus Approach

**DOI:** 10.1371/journal.pcbi.1000048

**Published:** 2008-04-04

**Authors:** Peng Wang, John Sidney, Courtney Dow, Bianca Mothé, Alessandro Sette, Bjoern Peters

**Affiliations:** 1 La Jolla Institute for Allergy and Immunology, La Jolla, California, United States of America; 2 Department of Biological Sciences, California State University-San Marcos, San Marcos, California, United States of America; Washington University, United States of America

## Abstract

The identification of MHC class II restricted peptide epitopes is an important goal in immunological research. A number of computational tools have been developed for this purpose, but there is a lack of large-scale systematic evaluation of their performance. Herein, we used a comprehensive dataset consisting of more than 10,000 previously unpublished MHC-peptide binding affinities, 29 peptide/MHC crystal structures, and 664 peptides experimentally tested for CD4+ T cell responses to systematically evaluate the performances of publicly available MHC class II binding prediction tools. While in selected instances the best tools were associated with AUC values up to 0.86, in general, class II predictions did not perform as well as historically noted for class I predictions. It appears that the ability of MHC class II molecules to bind variable length peptides, which requires the correct assignment of peptide binding cores, is a critical factor limiting the performance of existing prediction tools. To improve performance, we implemented a consensus prediction approach that combines methods with top performances. We show that this consensus approach achieved best overall performance. Finally, we make the large datasets used publicly available as a benchmark to facilitate further development of MHC class II binding peptide prediction methods.

## Author Summary

A critical step in developing immune response against pathogens is the recognition of antigenic peptides presented by MHC class II molecules. Since experiments for MHC class II binding peptide identification are expensive and time consuming, computational tools have been developed as fast alternatives but with inferior performance. Here, we carried out a large-scale systematic evaluation of existing prediction tools with the aim of establishing a benchmark for performance comparison and to identify directions that can further improve prediction performance. We provide an unbiased ranking of the performance of publicly available MHC class II prediction tools and demonstrate that the MHC class II prediction tools did not perform as well as the MHC class I tools. In addition, we show that the size of training data and the correct identification of the binding core are the two factors limiting the performance of existing tools. Finally, we make available to the immunology community a large dataset to facilitate the evaluation and development of MHC class II binding prediction tools.

## Introduction

The activation of CD4+ helper T cells is essential for the development of adaptive immunity against pathogens [1]–[4]. A critical step in CD4+ T cell activation is the recognition of epitopes presented by MHC class II molecules [5]. MHC class II molecules are heterodimers expressed on the surface of professional antigen presenting cells that bind peptide fragments derived from protein antigens [6]. X-ray crystallographic studies demonstrated that the MHC class II epitope binding site consists of a groove and several pockets provided by a β-sheet and two α-helices [7],[8]. Unlike class I, the class II binding groove is open at both ends. As a result, peptides binding to class II molecules tend to be of variable length, but typically between 13 and 25 residues.

A hallmark of the MHC class II binding peptide groove is that there are four major pockets. These pockets accommodate side-chains of residues 1, 4, 6, and 9 of a 9-mer core region of the binding peptide. This core region interaction largely determines binding affinity and specificity [9]. In addition, peptide residues immediately flanking the core region have been indicated to make contact with the MHC molecule outside of the binding groove, and to contribute to MHC-peptide interaction [10].

MHC class II molecules are highly polymorphic, and this polymorphism largely corresponds with differences along the peptide binding groove. However, the binding motifs derived for MHC class II molecules are highly degenerate, and many promiscuous peptides have been identified that can bind multiple MHC class II molecules [11]. Promiscuous peptides are a prime target for vaccine and immunotherapy and computational tools have been developed to facilitate systematic scanning for promiscuous peptides [12].

Computational prediction of MHC class II epitopes is of important theoretical and practical value, as experimental identification is costly and time consuming [13],[14]. The basis of a successful computational prediction is a sufficiently large set of high quality training data. There are several databases hosting MHC epitope related data such as SYFPEITHI [15], MHCBN [16], Antijen [17], FIMM [18], HLA Ligand [19] and our own project, the Immune Epitope Database (IEDB) [20],[21]. Information from those databases is, for the most part, extracted from the literature. These databases typically combine data from different sources and different experimental approaches, which can complicate the generation of consistent training and evaluation datasets.

The establishment of numerous MHC class II epitope databases has facilitated the development of a large number of algorithms aimed at predicting peptide binding to MHC molecules. Early works focused on finding peptide patterns and deriving motifs for MHC molecules [22]–[24]. With the accumulation of epitope data, more sophisticated algorithms were developed. Several methods have derived scoring matrices that evaluate the contribution to binding of different residues in a peptide based on quantitative binding data (ARB [25], SMM-align [26]). Others base similar scoring matrices on multiple peptide alignments (RANKPEP [27],[28]) or domain expert knowledge (SYFPEITHI method [15]). By combining the similarities of key residues forming the pockets of the binding groove with quantitative matrices derived from experiments, the TEPITOPE [29] algorithm can predict binding to MHC alleles for which no binding affinities were determined. Other machine learning algorithms that have been applied include hidden Markov models [30], evolutionary algorithms [31] and linear programming [32]. The MHC class II binding prediction problem has also been modeled with a distance function in a recently developed method PepDist [33]. In addition to the previously listed models that are directly interpretable, “black box” approaches, such as support vector machines [34] and artificial neural networks [35]–[37], have also been applied to MHC class II binding prediction with success.

Despite the large number of available prediction methods, computational prediction of MHC class II epitopes remains a challenging problem. It has been suggested that the prediction performance of class II algorithms is systematically inferior to that of MHC class I epitope prediction methods [25]. To assess the current state of the MHC class II binding predictions, we have here sought to establish a systematic and quantitative benchmark similar to our previous effort for MHC class I molecules [38]. We present a large dataset of unpublished MHC class II-peptide binding affinities that were experimentally determined under uniform conditions. We then proceed to evaluate a set of nine publicly available MHC class II prediction methods using this dataset and systematically compared their performance. Finally, we analyzed the ability of current methods to identify the binding cores of peptides and to predict T-cell responses from peptide sequences.

## Results

### Overview of MHC Class II Epitope Affinity Dataset and MHC Class II Binding Prediction Methods

We assembled a dataset of peptide binding affinities for various MHC class II molecules experimentally measured in our group (see Materials and Methods for details). Table 1 gives an overview of the dataset, encompassing a total of 10,017 experimentally determined peptide MHC II binding affinities. These data span a total of 16 human and mouse MHC class II types. The number of unique MHC-peptide affinities measured per type varies greatly, from 3,882 for HLA DRB1*0101, to only 39 for H-2-IE^d^. Compared to datasets publicly available on the IEDB and other MHC class II epitope databases, our new dataset expands the number of measured peptide-MHC class II interactions significantly for a large number of MHC class II molecules. For example, the number of peptides with known IC_50_ values for HLA DRB1*0101 was more than tripled with the addition of our new dataset.

**Table 1:** Overview of the MHC-peptide binding affinity dataset.

Organism	MHC class II types	Number of MHC-peptide affinities	
		New	Known^a^
Human	HLA-DRB1*0101	3882	1390
	HLA-DRB1*0301	502	817
	HLA-DRB1*0401	512	675
	HLA-DRB1*0404	449	233
	HLA-DRB1*0405	457	175
	HLA-DRB1*0701	505	424
	HLA-DRB1*0802	245	213
	HLA-DRB1*0901	412	174
	HLA-DRB1*1101	520	522
	HLA-DRB1*1302	289	242
	HLA-DRB1*1501	520	491
	HLA-DRB3*0101	420	104
	HLA-DRB4*0101	245	203
	HLA-DRB5*0101	520	383
Mouse	H-2-IA^b^	500	225
	H-2-IE^d^	39	231

a Number of records in IEDB as of 12-04-2006.

The MHC class II binding prediction tools evaluated in this study are listed in Table 2. We included as many prediction methods as possible provided that they (1) can perform predictions for MHC class II types in our dataset; (2) were publicly available; and (3) did not specifically disallow the use of automated prediction retrieval scripts. A total of nine methods matched these criteria. A more detailed description of tested methods is provided in the Materials and Methods section.

**Table 2:** Overview of nine MHC class II peptide prediction methods tested with the new dataset.

Category	Method	MHC class II types^a^	Training dataset	Algorithm
Matrix based	ARB	16 (16)	IEDB	Average relative binding (ARB) matrix
	PROPRED	51 (11)	TEPITOPE	Pocket profile
	SVMHC	51 (11)	TEPITOPE	Pocket profile
	SYFPEITHI	6 (6)	SYFPEITHI	Position specific scoring matrices
	RANKPEP	46 (16)	MHCPEP	Position specific scoring matrices
	SMM-align	17 (16)	IEDB SYFPEITHI	Stabilized matrix
Machine Learning based	SVRMHC	6 (5)	AntiJen	Support vector machine regression
	MHC2PRED	21 (15)	MHCBN JenPep	Support vector machine
Multivariate regression	MHCPRED	10 (6)	JenPep	Quantitative structure activity relationship (QSAR) regression

a Number of MHC class II types covered by a prediction method. The number in parentheses is the number of MHC class II types also in our dataset.

### Performance Evaluation of Publicly Available Prediction Tools

The binding predictions for peptides in our affinity dataset were extracted from the MHC class II binding prediction tools with custom scripts (see Materials and Methods for details). From the experimental data, peptides were classified into binders (IC_50_<1000 nM) and nonbinders (IC_50_≥1000 nM) based on measured affinities. The performance of the prediction methods was then measured by ROC curves (see Materials and Methods for details). Since the new dataset was never published before, it is equivalent to a blind test. An important exception is the ARB method. Since it was developed at IEDB and was constantly updating with new data, its performance was instead evaluated via 10-fold cross validation.

**Figure 1:**
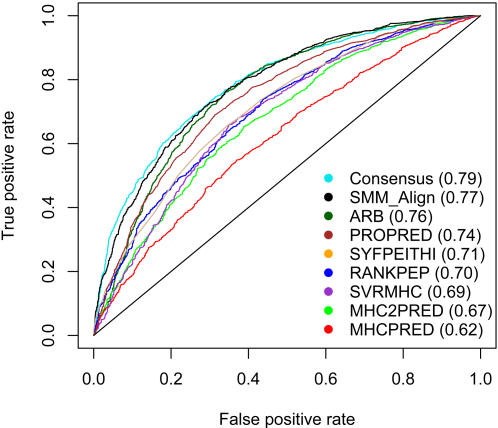
Performance of nine MHC class II prediction methods using HLA DRB1*0101 as an example. Prediction results for eight methods for HLA DRB1*0101 are shown in the ROC curve. The curves were generated by plotting the true positive rate (*y*-axis) against the false positive rate (*x*-axis). The AUC values for corresponding ROC curves were shown in parentheses.

Table 3 shows the prediction performance of the various methods in terms of area under ROC curve (AUC). The ROC curves for tested methods were also plotted in Figure 1 using HLA DRB1*0101 as an example. SVMHC was not evaluated separately, since it implements the same TEPITOPE matrices utilized by PROPRED. When overall performance is assessed by averaging across all available MHC class II molecules SMM-align and PROPRED are associated with the best AUC value (0.73). The ARB method has the third best performance with an average of AUC of 0.71. When performance on individual MHC class II molecule is examined, the ARB, PROPRED or SMM-align perform best for all but the H-2 IEd molecule, for which RANKPEP gives the best result.

**Table 3:** Performance of various MHC class II prediction methods^a^.

MHC class II type	Number of peptides	ARB	MHC2PRED	MHCPRED	PROPRED	RANKPEP	SMM-align	SVRMHC	SYFPEITHI	Consensus
DRB1*0101	3882	0.76	0.67	0.62	0.74	0.70	0.77	0.69	0.71	0.79
DRB1*0301	502	0.66	0.53		0.65	0.67	0.69		0.65	0.72
DRB1*0401	512	0.67	0.52	0.60	0.69	0.63	0.68	0.66	0.65	0.69
DRB1*0404	449	0.72	0.64		0.79	0.66	0.75			0.80
DRB1*0405	457	0.67	0.51		0.75	0.62	0.69	0.62		0.72
DRB1*0701	505	0.69		0.63	0.78	0.58	0.78		0.68	0.83
DRB1*0802	245	0.74	0.70		0.77		0.75			0.82
DRB1*0901	412	0.62	0.48			0.61	0.66			0.68
DRB1*1101	520	0.73	0.60		0.80	0.70	0.81		0.73	0.80
DRB1*1302	289	0.79	0.54		0.58	0.52	0.69			0.73
DRB1*1501	520	0.7	0.63		0.72	0.62	0.74	0.64	0.67	0.72
DRB3*0101	420	0.59					0.68			
DRB4*0101	245	0.74	0.61			0.65	0.71			0.74
DRB5*0101	520	0.7	0.59		0.79	0.73	0.75	0.63		0.79
IAB	500	0.8	0.56	0.51		0.74	0.75			0.86
IED	39			0.53		0.83				
Mean		0.71	0.58	0.58	0.73	0.66	0.73	0.65	0.68	0.76
Min		0.59	0.48	0.51	0.58	0.52	0.66	0.62	0.65	0.68
Max		0.8	0.70	0.63	0.80	0.83	0.81	0.69	0.73	0.86

a Performance is measured in terms of AUC as described in Materials and Methods. Evaluation of ARB was carried out via 10-fold cross validation. Evaluation of the rest of the methods were done as blind tests.

Since we restrict our testing to publicly available tools, it is important to point out that the methods were trained on different datasets (Table 2). Some databases such as MHCPEP only include positive binding data and the lack of nonbinders would be expected to negatively impact some methods that require negative training data. Two of the top performing methods (SMM-align and ARB) utilize the IEDB dataset, confirming that the size of the training set maybe an important factor contributing to better performance. PROPRED is among the most accurate MHC class II binding prediction methods, despite being based on the TEPITOPE method developed over eight years ago. The good predictive power of TEPITOPE demonstrates the validity of its approach, based on pocket information derived from MHC class II structures and quantitative peptide binding profiles.

The cutoff of 1000 nM to classify peptides into binders and non-binders was chosen following an expert immunologist's recommendation for an immunologically relevant threshold, but it is still somewhat arbitrary. To further our analysis in a systematic fashion, we varied the cutoff from 50 nM to 5000 nM. The changes in cutoffs enable us to evaluate performances of binding prediction to identify peptides with different affinities. A cutoff of 50 nM focuses on identifying strong binders, while a cutoff of 5000 nM will identify all including very weak binders. The results of the evaluation using different cutoffs are shown in Dataset S1. For MHC molecules with large number of binding affinities (such as HLA DRB1*0101), varying the cutoff has little impact on the AUC values. For datasets with smaller number of binding affinities (such as H-2-IE^d^), the change in AUC values is more significant. Despite the variations in AUC values introduced by different cutoffs, the relative performance of different methods remains largely the same, suggesting our conclusion of different methods' performance is not strongly dependent on the cutoff used to decide binders.

### Existing Tools Lack Consistency in Identifying the 9-Mer Core Interacting with the Binding Groove of the MHC Class II Molecule

A key difference between MHC class I and class II molecules is that the binding groove of class II molecules is open at both ends [7],[8]. As a result, the length of peptides binding class II molecules can vary considerably, and typically range between 13 and 25 amino acids long. Thus, a requisite for all MHC class II binding prediction approaches is the capacity to identify within longer sequences the correct 9-mer core residues that mediate the binding interaction [9]. All methods included in our study explicitly predict cores when they predict MHC class II binding peptides. They either predict binders as 9-mer peptides, or clearly state in prediction the location of the predicted 9-mer core.

We next analyzed whether the various class II prediction tools can accurately identify the 9-mer cores of a binding peptide. We extracted MHC-peptide complex structures from the Research Collaboratory for Structural Bioinformatics (RCSB) Protein Data Bank (PDB). A total of 29 structures associated with 14 different MHC class II molecules were identified (Table 4). For each method we compared the predicted cores with the true cores extracted from crystal structures. The results are shown in Table 5. The PROPRED method based on TEPITOPE was associated with the best performance, with all the predicted cores matching the cores determined by PDB structures. This is in good agreement with the fact that TEPITOPE is directly based on experimental assays. SYFPEITHI is the second best method with an accuracy of 0.9. However, this result should be interpreted with caution since seven of the nine correctly predicted peptides are documented in the SYFPEITHI database. Apart from PROPRED and SYFPEITHI, the method most effective in predicting binding affinity (SMM-align) is also the method with highest accuracy in predicting 9-mers cores, with an accuracy of 0.625. RANKPEP and SVRMHC come next with accuracies about 0.55. The remaining three methods had limited success (21–25%), although they still perform above random prediction (the probability to randomly guess the right core for a 15-mer peptide is 1 out of 7 or 0.143). Overall, these data suggest that correctly aligned cores contribute to the superior performance of PROPRED and SMM-align, and that there is substantial room to improve the quality of the core predictions of other methods.

**Table 4:** MHC class II structures used to evaluate the performance of different MHC class II epitope prediction methods.

Core	Peptide	Chain	PDB ID	MHC class II type
PFPQPELPY	LQPFPQPELPY	C	1S9V	DQB1*0201
EALYLVCGE	LVEALYLVCGERGG	C	1JK8	DQB1*0302
LPSTKVSWA	EGRDSMNLPSTKVSWAAVGGGGSLVPRGSGGGG	C	1UVQ	DQB1*0602
MRMATPLLM	PVSKMRMATPLLMQA	C	1A6A	DRB1*0301
FKGEQGPKG	AGFKGEQGPKGEPG	E	2FSE	DRB1*0101
IGILNAAKV	GELIGILNAAKVPAD	C	1KLG	DRB1*0101
VIPMFSALS	PEVIPMFSALSEGATP	C	1SJE	DRB1*0101
WRFLRGYHQ	GSDWRFLRGYHQYA	C	1AQD	DRB1*0101
YSDQATPLL	AAYSDQATPLLLSPR	C	1T5W	DRB1*0101
YVKQNTLKL	PKYVKQNTLKLAT	C	2G9H	DRB1*0101
MRADAAAGG	AYMRADAAAGGA	E	2SEB	DRB1*0401
YVKQNTLKL	PKYVKQNTLKLAT	C	1J8H	DRB1*0401
VHFFKNIVT	ENPVVHFFKNIVTPR	C	1BX2	DRB1*1501
FKNIVTPRT	NPVVHFFKNIVTPRTPPPSQ	C	1FV1	DRB5*0101
YHFVKKHVH	GGVYHFVKKHVHES	C	1H15	DRB5*0101
AQKAKANKA	FEAQKAKANKAVDGGGG	B	1LNU	IA^b^
MRMATPLLM	GSHSRGLPKPPKPVSKMRMATPLLMQALPMGSGSGS	C	1MUJ	IA^b^
SQAVHAAHA	RGISQAVHAAHAEI	B	1IAO	IA^d^
TQGVTAASS	GHATQGVTAASSHE	B	2IAD	IA^d^
IAPVFVLLE	YEIAPVFVLLEYVT	B	1ES0	IA^g7^
RHGLDNYRG	AMKRHGLDNYRGYS	P	1F3J	IA^g7^
DYGILQINS	STDYGILQINSRW	P	1IAK	IA^k^
HRGAIEWEG	GNSHRGAIEWEGIESG	P	1D9K	IA^k^
GGASQYRPS	HSRGGASQYRPSQRHGTGSGSGS	P	1K2D	IA^u^
IAYLKQASA	ADLIAYLKQASAKGG	B	1KTD	IEK
IAYLKQATK	ADLIAYLKQATKGGG	B	1KT2	IEK
IAYPKAATK	ADLIAYPKAATKF	E	1R5V	IEK
ITAFNDGLK	KKVITAFNDGLKGGG	B	1FNE	IEK
ITAFNEGLK	KKVITAFNEGLKGGG	B	1I3R	IEK

**Table 5:** Accuracy of MHC class II prediction methods for identifying epitope core regions.

MHC class II type	Known cores	Methods (Number of core regions identified correctly)							
		PROPRED	SMM-align	RANKPEP	ARB	MHCPRED	MHC2PRED	SVRMHC	SYFPEITHI
DQB1*0201	1	NA	NA	0	NA	NA	0	NA	NA
DQB1*0302	1	NA	NA	0	NA	NA	0	NA	NA
DQB1*0602	1	NA	NA	0	NA	NA	NA	NA	NA
DRB1*0101	6	6	5	5	4	1	2	3	6
DRB1*0301	1	1	1	1	0	NA	0	NA	1
DRB1*0401	2	2	1	1	0	0	2	0	1
DRB1*1501	1	1	1	1	0	NA	0	1	1
DRB5*0101	2	2	1	0	0	NA	0	2	NA
IA^b^	2	NA	1	2	0	0	0	NA	NA
IA^d^	2	NA	0	0	0	0	0	NA	NA
IA^g7^	2	NA	NA	0	NA	NA	1	NA	NA
IA^k^	2	NA	NA	1	NA	0	NA	NA	NA
IA^u^	1	NA	NA	0	NA	NA	NA	NA	NA
IE^k^	5	NA	NA	5	NA	3	NA	NA	NA
Accuracy (Correct/Total)	29	1.000 (12/12)	0.625 (10/16)	0.552 (16/29)	0.250 (4/16)	0.211 (4/19)	0.250 (5/20)	0.545 (6/11)	0.900 (9/10)

### Predicting T Cell Activation from Peptide Sequences with Existing MHC Class II Epitope Prediction Tools

The ultimate goal of MHC binding peptide prediction is to identify epitopes that activate T cells. Recognition of a peptide bound to an MHC molecule by a T cell receptors is the critical step in this activation, and binding of peptide to the MHC molecule is obviously a necessary requirement [39]. In a separate study carried out in our lab, a set of 664 peptides overlapping the LCMV proteome were tested for their abilities to promote H-2 IA^b^ specific IFN gamma production from CD4+ T cells in splenocytes from previously LCMV infected mice (manuscript in preparation). These peptides provided an ideal test set to evaluate MHC class II binding predictions as a tool to identify peptides that trigger an immune response.

**Figure 2:**
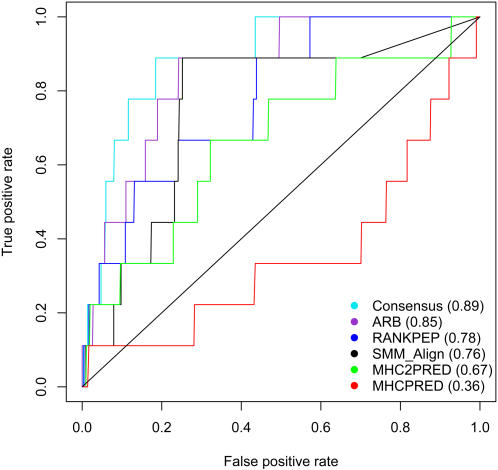
The performance of various MHC class II binding prediction approaches to identify CD4+ T cell epitopes. ROC curves are generated from the predictions made by five MHC class II peptide binding prediction methods on the LCMV CD4+ T cell activation data. The AUC value for each method is shown in parentheses.

For each of the 664 peptides, we obtained H-2 IA^b^ binding predictions from the five methods in our study that cover H-2 IA^b^ following exactly the same procedures as predictions of simple binding. We then evaluated the methods' performance in predicting which peptides triggered an immune response. The ROC curves quantifying the performance of each method are shown in Figure 2. The Consensus method is the best performing methods with AUC of 0.89±0.05. ARB is the second best performing method with an AUC of 0.85±0.05. SMM-align and RANKPEP have similar performance with AUC about 0.76±0.08 and 0.78±0.07, respectively. MHCPRED and MHC2PRED do not perform as well, with AUC values of about 0.67±0.12 and 0.36±0.1 (standard deviations calculated by bootstrapping with replacement). Except MHCPRED, every other method's performance in this evaluation compared favourably to that in predicting peptide binding. Overall, the ranking of prediction performances is well in concert with that for predicting peptide binding, specifically when taking into account the high standard deviations of AUC values. These large standard deviations are due to the limited number of positive datapoints in the set utilized.

To further analyze the performance of the T cell activation prediction, we classified peptides into predicted binders and non-binders. Since different methods produce scores on different scales, we adopt a rank based classification in that we classify the top 10% highest scoring peptides as binders. We then calculated sensitivity and positive predictive value (PPV) for each method (Table 6). These two measures were chosen since we are primarily interested in identifying T cell activating peptides while minimizing the number of false positive predictions. In our system, sensitivity is the percentage of peptides activating T cells predicted to be binders and PPV is the percentage of predicted binders that actually activate T cells. The data in Table 6 show that results of these two measures are largely consistent with the AUC results. Methods with high AUCs tend to have high PPV and sensitivity. Only the consensus method has sensitivity above 50%, indicating that 5 out of 6 methods missed more than half of the T cell activating peptides when top 10% ranked peptides are classified as binders. In addition, the consensus method also had the highest PPV value of 9.4%, making it again the best prediction method by this measure. The overall low PPV values are expected, as many peptides that are capable of binding MHC are not recognized by T cells following a natural infection, due to other factors such as peptide processing and the available T cell repertoire.

**Table 6:** Sensitivity and positive predictive value for predicting T cell activation.

	ARB	MHC2PRED	MHCPRED	RANKPEP	SMM-align	Consensus
Sensitivity	4/9 (44.4%)	2/9 (22.2%)	1/9 (11.1%)	3/9 (33.3%)	2/9 (22.2%)	6/9 (66.7%)
Positive predictive value	4/64 (6.2%)	2/64 (3.1%)	1/64 (1.6%)	3/64 (4.7%)	2/64 (3.1%)	6/64 (9.4%)

### Improving MHC Class II Binding Prediction with a Consensus Approach

Our evaluation of prediction performance suggests that in all cases there is clearly room for improvement, and that no single method is dominantly better than all others. Motivated by the success of a consensus prediction approach to map MHC class I epitopes in vaccinia virus [40], we implemented the same approach for MHC class II binding predictions. This consensus approach is based on calculating the median rank of the top three predictive methods for each MHC class II molecule (see materials and methods for details).

The consensus prediction performance is shown in the last column of Table 3 for the 14 MHC alleles for which three or more predictions were available. For ten of these fourteen alleles, the consensus method gives similar or higher performance than the best individual method. For each of the remaining four datasets, a single prediction performs better (i.e., ARB for DRB1*1302, SMM-align for DRB1*1101 and DRB1*1501, and PROPRED for DRB1*0405). In terms of overall performance across all molecules in our dataset, the consensus method outperforms all individual MHC class II prediction methods.

### Availability

The MHC-peptide affinity, MHC-peptide structure and T cell activation datasets are available as supplemental material at http://mhcbindingpredictions.immuneepitope.org/MHCII. These data are presented in this paper for immediate access by the immunology and bioinformatics community. We are currently in the process of depositing these data into the IEDB, making them available through the epitope informatics framework of the IEDB.

## Discussion

In this study we have presented a comprehensive dataset for the systematic evaluation of MHC class II peptide binding prediction methods. This dataset consists of three components. The first component is a large set of 10,017 quantitative peptide-binding affinities for 16 MHC class II types that significantly expands the amount of publicly available data. These data were generated under identical experimental conditions and comprise affinities for binders as well as non-binders. The second component is a set of non-redundant structures of MHC class II molecules complexed with peptide ligands compiled from the PDB. This set of structures provided a “gold standard” for evaluating the ability of prediction methods to locate the 9-mer core of epitopes. The last component is a set of 664 peptides that has been tested experimentally to determine their ability to stimulate CD4+ T cells from widely utilized C57BL/6 (H-2b) strain of laboratory mice. Together, these datasets serve as a benchmark set to facilitate the development and testing of algorithms for predicting peptide binding to MHC as well as T-cell responses.

Several previous studies have compared the performances of various MHC class II binding prediction methods [41]–[43]. The Borras-Cuesta study [43] from 2000 only had a limited number of peptides, alleles and methods to compare. The two recent studies were published after we finished our initial comparison. Gowthaman et al [42] compared six commonly used method with data spanning seven MHC class II alleles. However, their evaluation dataset comprised only 179 peptides, limiting the significance of their results. Rajapakse et al [41] compared their multi-objective evolutionary algorithms (MOEA) with five other algorithm using two datasets. The first dataset consisted of 1 training and 10 testing datasets on HLA DRB1*0401 assembled from different sources. The second dataset was extracted from the IEDB and comprised more than 5,000 peptides covering 16 MHC class II alleles. We couldn't include MOEA in our comparison since it is not publicly available at the moment. Despite the difference in datasets used in comparison, their conclusion is consistent with ours in that SMM-align, TEPITOPE and ARB are the better performing methods.

We have carried out a comprehensive unbiased evaluation of existing MHC class II epitope prediction algorithms using these datasets. Except binding prediction for ARB, all the other MHC class II prediction algorithms are evaluated in a completely blinded fashion. In our analysis, the better performing methods proved to be those that are based on quantitative matrices extended by method specific features. For example, SMM-align is the only method tested that considers the contribution of residues outside of the binding groove, and TEPITOPE is the only method whose matrices are based on experiments aimed to determine individual amino acid's contribution to binding. Merely using quantitative matrices alone is not sufficient to ensure good performance, since pure position specific scoring matrix based methods such as RANKPEP and SYFPEITHI do not perform as well.

One potential reason for the differential performance of various methods is the likely different number of data points utilized by the various methods in the training stage. In this respect, we anticipate that the datasets described herein, and now made publicly available, could be utilized to retrain several of the methods and further increase their performance.

Despite the large number of existing MHC class II epitope prediction methods, the best performance is generally not as good as that for MHC class I methods. Indeed, it is notable that the majority of methods examined in the present study have also been employed to make predictions for MHC class I peptide binding, and almost invariably their performance is appreciably better in the context of class I [38]. For example, when SMM [44] was applied to predict epitopes for several MHC class I molecules, it achieved an average AUC of 0.874, which is substantially higher than that for class II (0.783).

In an attempt to identify what limits the performance of MHC class II binding prediction, we tested the ability of prediction methods to identify the 9-mer peptide cores revealed in crystal structures of MHC-peptide complexes. Except for PROPRED and SYFPEITHI, the methods examined performed poorly, suggesting that difficulties in identification the correct binding core contribute to the inferior performance of class II binding prediction. It is noteworthy that the two methods with the best core predictions do not take all positions of a peptide into account when making binding predictions, but rather focus on anchor positions in the peptide. This may explain why especially the ARB method performs much poorer in the core identification rather than the binding predictions: It treats all positions in the peptide identically and relies on automated peptide alignments to derive an overall peptide profile. While this inclusion of weakly interacting positions can be an advantage to predict overall peptide binding, it may lower the accuracy when picking the correct core.

In an attempt to improve upon the prediction performance realized by individual prediction tools, we implemented a consensus approach for class II binding predictions. The consensus approach was found to clearly outperform each individual prediction approach when measured over the entire dataset, and provided the best predictions for 10 out of 14 molecules. This shows that the consensus approach is just as useful for MHC class II peptide binding prediction as its recent successful application for MHC class I molecules [40]. In a smaller study addressing 3 different prediction methods in the context of a single DR type, Mallios previously came to a similar conclusion [45].

Other types of meta approaches have been successfully applied to MHC binding prediction. For example, Mallios [46] has used an iterative stepwise discriminant analysis meta-algorithm to successfully classify binders and non-binders for HLA-DR1. Stern and co-workers effectively used a two-dimensional dot plot to combine the prediction results of SYFPEITHI and TEPITOPE [47]. Finally, Trost et al [48] have reported achieving greater accuracy in MHC class I binding predictions by combing results from multiple prediction tools. Compared to these methods, our median rank approach does not depend on the absolute values of scores and it has exceptional scalability since typical sorting algorithms have running times proportional to *n* log *n* where n is the number of cases needed to be sorted. Overall, it is astonishing that the systematic use of consensus predictions comes rather late (see Mallios [45],[46]) to the problem of MHC peptide binding since consensus approaches have for quite some time proven their superiority in a number of fields, notably protein structure prediction [49].

In any case, it is also likely that the remarkable increase in performance obtained by the use of the consensus approach hinges on the fact that it combines information derived from methods trained on large numbers of data points with methods incorporating structural considerations leading to effective core predictions. We are currently working on development of algorithms specifically combining these two different features.

We also tested the ability of MHC class II binding prediction methods to predict a peptide's ability to activate CD4+ T cells. Most of the methods were associated with good performance. This was somewhat surprising since T cell activation is a multi-step process where multiple signals are needed for successful activation [50]–[52]. In addition, a peptide that binds well to MHC molecules is not necessarily a good stimulator for T-cell response as different amino acids are interacting with T cell receptor. It is important to point out that the performance was based on a set of 664 peptides of which only 9 activated CD4+ T cells. The limited number of positive cases makes the ROC curve jagged and the AUC values calculated less robust. Despite the encouraging AUC values achieved by several methods, it is still necessary to test a large number of peptides to identify most of the T cell activating peptides. In addition, all those methods still have high numbers of false positives peptides that are predicted binders but will not activate T cells. Since experimental efforts to test T cell activation are even more time consuming than testing peptide-MHC binding, significant efforts are needed to develop tools that can identify T cell activating peptides with high sensitivity and specificity.

In conclusion, we have presented a set of benchmarks to facilitate the evaluation and development of MHC class II binding predictions. While several good methods are available, these do not reach the performance of those for MHC class I molecules. We have shown that a simple and robust consensus approach can improve the prediction performance for the great majority of the MHC class II molecules tested. Finally, we speculate that novel approaches that capture distinct features of MHC class II peptide interactions could lead to more successful predictions than the current approaches, which are commonly developed as extensions of MHC class I predictions.

## Materials and Methods

### Peptide Synthesis

Peptides utilized for the assessment of MHC binding, antigenicity and immunogenicity were purchased as crude material from Mimotopes (Minneapolis, MN and Clayton, Victoria, Australia), Pepscan Systems B.V. (Leylstad, Netherlands) or A and A Labs (San Diego, CA). Quality control analyses of crude syntheses were performed by mass spectrometry on randomly selected peptides. Peptides selected for additional deconvolution and HLA peptide binding assays were resynthesized by A and A as purified material. Peptides were purified to >95% by reversed-phase HPLC, and the purity assessed by amino acid sequence and/or composition analysis.

### Experimental Procedures to Measure MHC Class II Peptide Affinity

Quantitative assays to measure the binding affinities of peptides to purified soluble class II molecules are based on the inhibition of binding of a radiolabeled standard peptide. Binding assays were performed essentially as described previously [13],[53]. Briefly, 0.1–1 nM radiolabeled peptide was coincubated for 2 days at room temperature with 1 µM to 1 nM purified MHC in the presence of a cocktail of protease inhibitors. Following a two-day incubation, the amount of MHC bound labelled peptide was determined by capturing MHC/peptide complexes on LB3.1 antibody coated Lumitrac 600 microplates (Greiner Bio-one, Longwood, FL), and measuring bound cpm using the TopCount microscintillation counter (Packard Instrument Co., Meriden, CT). Individual peptides were typically tested in 3 or more independent experiments for its capacity to inhibit the binding of the radiolabeled peptide. The concentration of peptide yielding 50% inhibition of the binding of the radiolabeled peptide was calculated. Under the conditions used, in which [label]<[MHC] and IC_50_≥[MHC], the measured IC_50_ values are reasonable approximations of the true *K*_d_ values. The binding affinities are expressed in terms of IC_50_ and are capped at 50,000 nM, reflecting the experimental sensitivity threshold.

### Dataset of Binding Affinities Used in the Study

The assembled MHC class II peptide binding affinities are listed in Table 1. The peptide binding affinities for various MHC class II molecules were generated in the context of various projects currently ongoing in our laboratory. Because they have been recently generated, to the best of our knowledge, none of the binding affinities in this dataset has been previously published. This assessment was confirmed by comparing our dataset to publicly available records contained in the IEDB (Table 1) or elsewhere. There are total 10,017 measured affinities in our dataset spanning thirteen human and three mouse MHC class II types. Peptides for 114 proteins from 30 organisms were synthesized and tested. While peptide sizes ranged form 9 to 37 amino acids, the vast majority of the measured affinities are for 15-mers (9,632 out of 10,017). The present dataset is currently in the process of being deposited in the IEDB.

### PDB Structures of MHC Class II and Epitope Complexes

Structures of MHC class II were retrieved from the Protein Data Bank with a keyword search (using keyword “MHC class II”). The retrieved structures were then examined to select complexes have epitopes with at least 9 amino acids. In addition, the structures were examined to identify entries with identical MHC and binding peptide sequences. For duplicated structures of the same MHC and epitope, we retained the structure with the highest resolution. The final dataset contains 29 non-redundant structures.

### MHC Class II Binding Prediction Tools Evaluated in This Study

The eight MHC class II binding prediction tools evaluated in this study are listed in Table 2. Five of the prediction methods are based on various scoring matrices. The method developed at IEDB utilizes the Average Relative Binding (ARB) matrix [25]. PROPRED [54] and SVMHC [55] are web servers based on TEPITOPE's pocket profile [29]. Both SYFPEITHI [15] and RANKPEP [28] use position specific matrices. Another matrix based approach, SMM-align [26], utilizes the stabilized matrix method (SMM [44]), but introduces a novel step to identify peptide binding cores, which makes it applicable to MHC class II predictions. Two of the methods, SVRMHC [56] and MHC2PRED (http://www.imtech.res.in/raghava/mhc2pred/index.html), apply support vector machine or support vector regression to predict epitopes. Finally, MHCPRED is a quantitative structure activity relationship (QSAR) regression based method [57].Three of the nine methods, ARB, MHC2PRED and SMM-align, give predictions in terms of the quantitative affinity of a peptide for a MHC class II molecule. The predictions of the other six methods are given as a score which is not directly translatable into an affinity of peptide-MHC binding.

In terms of the number of MHC class II types covered, the two TEPITOPE based methods (PROPRED and SVMHC) have the broadest coverage with 51 types, 11 of which also appear in our dataset. The next most comprehensive method is RANKPEP which covers 46 types, 16 of which overlap with our dataset. ARB, MHC2PRED and SMM-align make predictions for about 20 MHC class II types and the majority of the types (15 to 16) also appear in our dataset. The three remaining methods (MHCPRED, SVRMHC and SYFPEITHI) have less coverage, as they only predict peptide binding for 5 to 6 MHC class II types in our dataset.

Table 2 also lists the dataset used by each method to train their predictive models. Training on larger sets of data would be expected to yield better performance when tested on independent new data. In this context, the IEDB has HLA-DRB1*0101 binding information for 1390 peptides, AntiJen for 730, and MHCBN for 588. By contrast, SYFPEITHI lists only 42 entries for HLA-DRB1*0101. Thus the ARB and SMM-align methods which use data from the IEDB, had access to the largest training set compared to other methods, while the SYFPEITHI method had access to the smallest dataset.

### MHC Class II Epitope Prediction with External Tools

We identified eight publicly available MHC class II prediction tools through literature search and the IMGT link list at http://imgt.cines.fr/textes/IMGTbloc-notes/. For each tool, we mapped the MHC types for which predictions could be made to the four-digit HLA nomenclature (e.g., HLA-DRB1*0101). If this mapping could not be done exactly, we left that type/tool combination out of the evaluation. For example, HLA-DR4 could refer to HLA-DRB1*0401, DRB1*0402 etc, which do have distinct binding specificities.

For the ARB evaluation, the 10-fold cross validation results stored at IEDB was used to estimate performance since ARB was trained on datasets overlapping with the one used in this study. For the other seven tools in the evaluation, we wrote python script wrappers to automate prediction retrieval. For the SYFPEITHI prediction, we patched each testing peptide with three Glycine residues at both ends before we submitted it for prediction. This was recommended by the creators of SYFPEITHI method to ensure that all potential binders are presented to the prediction algorithm. For all other methods, the original testing peptides were submitted directly for prediction. Peptide sequences were sent to the web servers one at a time and predictions were extracted from the server's response. To assign a single prediction for peptides longer than nine amino acids in the context of tools predicting the affinity of 9-mer core binding regions, we took the highest affinity prediction of all possible 9-mers within the longer peptide as the prediction result.

### Consensus Approach to Predict MHC Class II Binding Peptides

For each MHC class II molecules whose binding can be predicted by three or more algorithms, we employed the following approach to generate a consensus prediction. First, we selected the top three methods that give the best performance. For each method, the tested peptides are ranked by their scores with higher ranks for better binders. For each tested peptide, the three ranks from different methods are then taken and the median of the three is calculated. This median rank is taken as the consensus score.

### Performance Measure of External Tools

Receiver operating characteristic (ROC) curves [58] were used to measure the performance of MHC class II binding prediction tools. For binding assays, the peptides were classified into binders (experimental IC_50_<1000 nM) and nonbinders (experimental IC_50_≥1000 nM), which was determined as a practical cutoff in a previous study [59]. For CD4+ T cell activation assays, the peptides were classified into T-cell epitopes (experimental SFC count≥100) or non-epitopes (experimental SFC count <100). For a given prediction method and a given cutoff for the predicted scores, the rate of true positive and false positive predictions can be calculated. An ROC curve is generated by varying the cutoff from the highest to the lowest predicted scores, and plotting the true positive rate against the false positive rate at each cutoff. The area under ROC curve is a measure of prediction algorithm performance where 0.5 is random prediction and 1.0 is perfect prediction. The plotting of ROC curve and calculation of AUC are all carried out with the ROCR [60] package for R [61].

### LCMV Epitope Identification

C57BL/6 (H-2^b^) mice were purchased from The Jackson Laboratory (Bar Harbor, ME), and infected intraperitoneally with 2×10^5^ PFU of LCMV Armstrong (i.p.). Spleens were harvested eight days post infection, and IFN-γ ELISPOT assays were performed as previously described [62] using CD4+ T cells isolated with anti-CD4+ magnetic beads (Miltenyi Biotech Inc., Auburn, CA). Experimental values were expressed as the mean net spots per million CD4+ cells ±SD for each peptide pool or individual peptide. For the initial screening of the 83 pools, responses against each pool were considered positive if a) the number of spot forming cells (SFCs) /10^6^ CD4+ T cells exceeded the absolute value of the mean negative control wells (effectors plus APCs without peptide) by two-fold, b) the value exceeded 200 SFCs/10^6^ CD4+ cells and c) these conditions were met in at least two replicate independent experiments. Positive pools were deconvoluted into their eight individual components and tested again, to determine which individual peptides were responsible for the pooled IFN-γ response. Responses against individual peptides were considered positive if they exceeded the threshold of the mean negative control wells (effectors plus APCs without peptide) by at least 2 standard deviations and exceeded a threshold of 200 SFCs/10^6^ CD4+ cells.

## Supporting Information

Dataset S1 AUC values for the tested MHC class II binding prediction methods using different cutoffs. The cutoffs for binders were varied from 50 nM to 5000 nM.
(0.03 MB XLS)
